# Medical use and costs for native fathers and children from transnational marriage families in Taiwan from 2004 to 2017

**DOI:** 10.3389/fpubh.2022.870859

**Published:** 2022-10-04

**Authors:** Yi-Lung Chen

**Affiliations:** ^1^Department of Healthcare Administration, Asia University, Taichung, Taiwan; ^2^Department of Psychology, Asia University, Taichung, Taiwan

**Keywords:** medical cost, medical use, transnational marriage, immigrant family, minority, healthcare utilization

## Abstract

**Objectives:**

This is the first study to examine health care utilization in terms of medical use and costs in native fathers and children from transnational families.

**Methods:**

Taiwan National Health Insurance Research Database was used to compare the annual medical use and inflation-adjusted medical cost for ambulatory care from 2004 to 2017 between native fathers and children from transnational and native families.

**Results:**

Native fathers from transnational families had lower annual medical use (−0.23 visits) but higher total medical costs (New Taiwan dollars, NT$, 966), especially in dialysis and psychiatry, compared with those from native families. Unlike fathers from transnational families, their children were observed to consistently have lower medical use (−1.35 visits) and costs (NT$ −636), compared with those from native families.

**Conclusions:**

There was different medical use and costs in transnational marriage families, possibly as a result of features in transnational marriage families. These findings provide insight for future health care policies to address the different health care utilization by exploring the unmet needs and barriers relating to children and fathers from transnational families.

## Highlights

- Although some studies reported a lower health care utilization rate in terms of medical expenditures or health care consumption for specific diseases in immigrants, none focused on fathers and children from immigrant marriage families.
- This is the first study to examine medical use and costs in fathers and children from transnational families, and it demonstrated different medical use and costs between native fathers and children from transnational families and native families.
- Further studies are warranted to exploring whether there are unmet needs or barriers relating to the different health care utilization in children and fathers from transnational families.

## Introduction

Although the World Health Organization set up health equality as the primary target in 2000 (1), different health care utilization was observed in different socioeconomic and ethnic minorities, such as individuals with low socioeconomic status, ethnic minority status and immigrants (2–4). However, transnational marriage families, a special societal minority, has not received comprehensive population-wide analysis of medical use and costs so far. Transnational marriage brokerage (also called the mail-order bride phenomenon) is a commercialized marriage through international marriage brokering agencies; most often women from less developed countries marry men in more developed countries. Although this phenomenon occurs worldwide, it seems more common in East and Southeast Asia (5–7). In Taiwan, international marriages contributed to 15.3 to 23.8% of annual marriages from 2004 to 2017 (8), and newborns of international marriages accounted for 13.2 to 6.1% of all newborns in Taiwan from 2004 to 2018 (8). After the emergence of transnational marriages, several studies have examined the health equality for these East or Southeast Asian female marriage immigrants (9, 10); however, thus far, this attention has mainly been paid to female marriage immigrants, and their Taiwanese family members (husbands and children) have received much less attention. To the best of my knowledge, studies examining the profiles of native men with female marriage immigrants have been conducted using informant methods (i.e., information gathered by their female marriage immigrants, not self-report by their native-born husbands) (11–13), and no study has directly examined their health status and medical use and costs. There may be different health care utilization for these male spouses and children because men who cannot get married to local women need international marriage brokering agencies to get married instead, which may be the result of their socioeconomic vulnerabilities and interpersonal or health problems (5). Furthermore, owing to the lack of firm relationships in commercialized marriages and the differences in culture and language between couples in commercial marriage, fathers and children from such families may have more health problems than those from native families (13, 14).

Taiwan has a statutory health insurance system with obligatory national insurance, which makes it a privilege to explore medical use and costs in this special minority group. Taiwan launched a single-payer mandatory enrollment National Health Insurance Program in 1995, which is a single-payer system is publicly funded primarily through payroll-based premiums with copayment. The regular premiums are calculated based on the insured's salary, and are shared by the insured, the employer, and the government. Furthermore, the insurance premium and copayment will be subsidized or reduced for the disadvantaged groups (15). Taiwan's National Health Research Institutes established and continued to maintain the Taiwan National Health Insurance Research Database (TNHIRD) for public research purposes. The TNHIRD has almost full coverage (99.99%) of citizens and hospitals contracted in Taiwan, and the health care information in the TNHIRD includes primary care, hospital care, pharmaceutical care, home care, and dental care and linkable health-related databases, such as birth, death, and maternal and welfare datasets (16). The complete data on annual insured health care costs make it possible to identify medical use and costs across differing socioeconomic and ethnic minorities.

This study explored and compared the medical use and costs between native fathers and children from transnational families and fathers and children from native families. I did not compare marriage immigrant mothers and native mothers because marriage immigrant mothers did not have information on medical use and costs in the health insurance system before their international marriages. Furthermore, lower medical use is expected in immigrant mothers after they were enrolled in the National Health Insurance program because of the difficulties of access to health care services such as unfamiliarity, transportation and differences in language and culture (17).

## Methods

### Study sample

All datasets used in this study were from TNHIRD database. This study sample consisted entirely of liveborn children with complete information on the nationality of both parents from the Taiwan Birth Certificate Registration from 2004 to 2016 to ascertain native Taiwanese fathers from transnational families and native families. I restricted transnational marriage families to those in East or Southeast Asian countries, which were defined as China, Vietnam, Indonesia, Thailand, the Philippines, Malaysia, Myanmar and Cambodia. I did not include South Korea and Japan in East or Southeast Asian countries because the development index in these countries was close to or higher than that in Taiwan. Because the Taiwan Birth Certificate Registration dataset only contained the identity of mothers, a linkage to the Taiwan Maternal and Child Health Database with key variables of mother's id and the sex and birth year of was made to obtain complete identity on children and their fathers. The Taiwan Birth Certificate Registration database includes data on 99.78% of all births nationwide in Taiwan from 2004 to 2016 (18).

### Covariates

In this study, I used age (by the end of the study) and low-income family status as controlling covariates. Low-income family status was determined using the Low-Income and Middle-Low-Income Households dataset. Low-income family defined by the Taiwan government is that individuals whose family incomes evenly distributed to the number of family members is under the minimum living expense and household assets, which is adjusted once every 4 years based on the growth rate of consumer price index (19). In 2022, household assets is average movable property under 75,000 New Taiwan dollar (NT$) and an overall real estate value under 3.76 million NT$ for low-income family, and with average movable property under 112,500 NT$ and an overall real estate value under 5.64 million NT$ for middle-low-income family, respectively (20).

### Medical use and cost

I used a national health facility database, the Taiwan National Health Insurance Research Database, to examine participants' medical use and cost. Medical use was measured by the frequency and cost of care associated with ambulatory visits (including treatment with Western medicine, emergency care, community care, and home care). Although the Taiwan's National Health Insurance has covered the traditional Chinese medicine, I focused only on Western Medicine instead of traditional Chinese Medicine because traditional Chinese Medicine was limited to a certain area. All medical costs, comprising consultation fees, diagnostic fee, laboratory examination fee, medicine service fee, and medicine fee, were recorded in points and under the global budget floating and average value system, in which 1 point equaled 0.8347 to 1.0573 NT$. Because of the global budget payment system, the medical payments is settled by the end of every season in different bureaus of National Health Insurance Administration, thus the costs varied in different seasons by year and geographic regions from 2004 to 2017. On December 1, 2021, the exchange rate of NT$ against the US and Euro dollar was approximately 28 to 1 and 32 to 1, respectively (21). To address economic inflation, I used the latest consumer price index released by Directorate-General of Budget, Accounting and Statistics, Executive Yuan, Taiwan, with the reference year of 2020 as 1 to obtain inflation-adjusted medical cost.

I used the individual's average annual medical use and cost, which was medical use, and costs were further adjusted by the individual's follow-up time. Fathers were followed from January 1, 2004, until death or the end of study (December 31, 2017), whereas children were followed from their birth date until death or the end of the study.

### Statistical analysis

All statistical analyses were performed with SAS version 9.4 (SAS Institute, Cary, NC, USA). I compared demographic and low-income status between native fathers and children from transnational families and fathers and children from native families by using chi-square tests for categorical variables and independent *t*-tests for continuous variables.

To estimate the difference in average annual medical use, average inflation-adjusted medical cost per visit, and average annual inflation-adjusted medical cost in total and in the top leading medical specialties between native fathers and children from transnational families and fathers and children from native families, Wilcoxon rank-sum test and general linear models (GLMs) were used. For Wilcoxon rank-sum test, the median of medical use indexes was compared without adjustment for any covariates. For the GLM, the mean difference of medical use indexes was compared with adjustment for demographic and low-income status, and adjusted regression coefficients and 95% confidence intervals (CIs) were reported. If the 95% CI of adjusted regression coefficients contains the null value of 0, no difference was observed between groups. Finally, I used treemaps and arrow diagrams to depict the proportions and ranking of average annual inflation-adjusted medical cost in different medical specialties between native fathers and children from transnational families and fathers and children from native families.

## Results

### Difference of medical use and costs

This sample included 1,300,684 fathers and 1,763,330 children, 6.9% of whom were fathers and 6.3% of whom were children whose spouses and mothers were from East or Southeast Asia. Table 1 presents the difference in sociodegraphics and medical use and costs between native fathers from transnational families and fathers from native families. Native fathers from transnational families were older and more likely to have low-income status than fathers from native families. Generally, the average annual ambulatory medical use was approximately six to seven visits. Although I first observed that native fathers from transnational families had higher medical use than fathers from native families, after controlling for their age and low income, the opposite finding was observed; native fathers from transnational families had −0.23 (95% CI: −0.33 to −0.14) lower average annual ambulatory medical use than fathers from native families. In terms of average annual inflation-adjusted medical cost, native fathers from transnational families were significantly associated with $966 (95% CI: $791 to $1141) higher ambulatory medical care costs in total than fathers from native families. Given fewer average annual outpatient visit and higher average annual inflation-adjusted medical cost in native fathers from transnational families, they had higher average medical cost per visit than fathers from native families. Specifically, such higher medical costs were observed in most common medical specialties, including family medicine, general medicine, dialysis, surgery, orthopedics, urology, and psychiatry; however, lower medical costs were found in otorhinolaryngology, dermatology, and gastroenterology.

**Table 1:** Demographics, medical use and costs between fathers from transnational and native families.

**Variable**	**Fathers from native families**		**Native fathers from transnational families**		**Statistics**	
	***N*** = **1,211,001**		***N*** = **89,683**			
Age (year), mean (SD)	40.42 (6.04)		45.75 (7.40)		*P* < 0.001	
Low–income, *n* (%)	43,538 (3.60)		8,906 (9.93)		*P* < 0.001	
Cumulative person–year, sum	16,912,801		1,242,825		–	
	**Median (IQR)**	**Mean (SD)**	**Median (IQR)**	**Mean (SD)**	**Unadjusted median difference** ^a^	**Adjusted mean Difference**^b^ **B (95% CI)**
No. of average annual outpatient visit	5 (3, 8)	6.63 (14.20)	5 (2, 9)	7.04 (7.09)	*P* < 0.001	−0.23 (−0.14, −0.33)
Total annual average medical care costs (NT$)	3065 (1537, 5304)	5028 (24473)	3155 (1513, 6954)	7166 (32459)	*P* < 0.001	966 (791, 1141)
Average cost per visit (NT$)	596 (463, 741)	759 (1135)	632 (485, 890)	1018 (1562)	*P* < 0.001	103.4 (94.6, 112.2)
**Medical care in top 10 medical specialties (NT$)**					
Family medicine	217 (78, 507)	461 (910)	228 (67,634)	643 (1448)	*P* < 0.001	100 (93, 106)
General medicine	168 (51, 431)	442 (7875)	158 (37, 497)	598 (7294)	*P* < 0.001	65 (11, 120)
Dialysis	0 (0, 0)	131 (6630)	0 (0, 0)	553 (14177)	*P* < 0.001	273 (221, 324)
Surgery	80 (0, 279)	251 (2036)	90 (0, 323)	327 (2805)	*P* < 0.001	61 (46, 76)
Orthopedics	86 (0, 272)	248 (522)	72 (0, 278)	279 (694)	*P* < 0.001	19 (16, 23)
Urology	0 (0, 67)	249 (1614)	0 (0, 92)	376 (2734)	*P* < 0.001	29 (17, 41)
Psychiatry	0 (0, 0)	115 (1139)	0 (0, 0)	375 (3213)	*P* < 0.001	213 (204, 223)
Emergency department	0 (134, 383)	321 (901)	125 (0, 437)	416 (1093)	*P* < 0.001	89 (83, 96)
Rehabilitation	0 (0, 0)	179 (983)	0 (0, 0)	241 (1985)	*P* = 0.008	2 (−4, 10)
Otorhinolaryngology	268 (83, 656)	522 (2857)	134 (28, 445)	407 (865)	*P* < 0.001	−150 (−131, −170)
Dermatology	87 (20, 263)	261 (1553)	43 (0, 173)	220 (1710)	*P* < 0.001	−41 (−30, −52)
Gastroenterology	12 (0, 183)	354 (1683)	0 (0, 185)	409 (2336)	*P* < 0.001	−60 (−48, −72)

^a^Aanaysis was conducted using Wilcoxon rank–sum test.

^b^Adjusted for age and low–come status.

SD, Standard deviation; IQR, Interquartile range.

Fathers from native families serve as the reference group.

New Taiwan dollars (NT$).

On December 1, 2021, the exchange rate of NT$ against the US and Euro dollar was approximately 28 to 1 and 32 to 1, respectively, based on the Bank of Taiwan (https://rate.bot.com.tw/xrt/all/2021-12-01?Lang=en-US).

A similar analysis was conducted to determine the difference in medical use and costs between children from transnational families and children from native families (Table 2). Compared to children from native families, children from transnational families were more likely to be boys, older, and from low-income families. Generally, the average annual ambulatory medical use and the average annual inflation-adjusted medical cost were 20.84 visits and 12021 NT$ for children from native families and 16.98 visits and 9,842 NT$ for children from transnational families. Unlike their fathers, children from transnational families were consistently more likely to have lower medical use (adjusted regression coefficient: −3.16 and 95% CI: −2.04 to −4.28) and medical costs in total (adjusted regression coefficient: −2166 NT$ and 95% CI:−1714 to −2618) and in most medical specialties, except for family medicine, but no difference was observed in average medical cost per visit.

**Table 2:** Demographics, medical use and costs between children from transnational and native families.

**Variable**	**Children from native families**		**Children from transnational families**		**Statistics**	
	***N*** = **1,652,141**		***N*** = **111,189**			
Age (year), mean (SD)	6.33 (3.50)		6.66 (3.52)		*P* < 0.001	
Sex (boy), *n* %	854,713 (51.73)		57,868 (52.04)		*P* < 0.001	
Low–income, *n* (%)	62,993 (3.81)		11,045 (9.93)		*P* < 0.001	
Cumulative person–year, sum	10,446,715		739,436		–	
	**Median (IQR)**	**Mean (SD)**	**Median (IQR)**	**Mean (SD)**	**Unadjusted median difference** ^a^	**Adjusted mean Difference**^b^ **B (95% CI)**
No. of average annual outpatient visit	18 (12, 22)	20.84 (43.13)	16 (9, 20)	16.98 (18.32)	*P* < 0.001	−3.16 (−2.04, −4.28)
Total annual average medical care costs (NT$)	8729 (5712,11023)	12021 (73236)	6950 (4568, 8936)	9842 (45326)	*P* < 0.001	−2166 (−1714, −2618)
Average cost per visit (NT$)	474 (426, 543)	537 (731)	483 (427, 543)	540 (1201)	*P* = 0.102	−0.58 (−4.26, 3.1)
**Medical care in top 10 medical specialties (NT$)**					
Family medicine	307 (35, 1173)	1614 (20004)	365 (58, 1271)	1741 (23948)	*P* < 0.001	24 (18, 30)
General medicine	0 (0, 162)	199 (11558)	0 (0, 192)	294 (24393)	*P* = 0.031	60 (−15, 135)
Surgery	0 (0, 24)	68 (434)	0 (0, 35)	76 (429)	*P* = 0.062	−2 (−5, 1)
Pediatrics	4211 (2164, 7239)	3016 (28194)	2789 (1149, 5433)	2496 (27316)	*P* < 0.001	−673 (−500, −846,)
Otorhinolaryngology	579 (84, 1978)	916 (6233)	360 (0, 1448)	763 (1323)	*P* < 0.001	−221 (−184, −258)
Ophthalmology	155 (0, 396)	219 (521)	94 (0, 284)	176 (355)	*P* < 0.001	−60 (−57, −63)
Dermatology	61 (0, 229)	110 (411)	0 (0, 142)	81 (203)	*P* < 0.001	−36 (−35, −37)
Psychiatry	0 (0, 0)	58 (552)	0 (0, 0)	55 (541)	*P* < 0.001	−10 (−7, −13)
Rehabilitation	0 (0, 0)	706 (5417)	0 (0, 0)	713 (5417)	*P* < 0.001	−75 (−43, −107)
Emergency Department	343 (0, 1029)	411 (814)	282 (0, 975)	442 (810)	*P* = 0.044	−2 (−6, 2)
Pediatric Surgery	307 (35, 1173)	68 (628)	0 (0, 0)	57 (415)	*P* < 0.001	−13 (−8, −18)

^a^Aanaysis was conducted using Wilcoxon rank–sum test.

^b^Adjusted for sex. age and low–come status.

SD, Standard deviation; IQR, Interquartile range.

Children from native families serve as the reference group.

New Taiwan dollars (NT$).

On December 1, 2021, the exchange rate of NT$ against the US and Euro dollar was approximately 28 to 1 and 32 to 1, respectively, based on the Bank of Taiwan (https://rate.bot.com.tw/xrt/all/2021-12-01?Lang=en-US).

### Proportion and ranking of medical costs in different medical specialties

**Figure 1:**
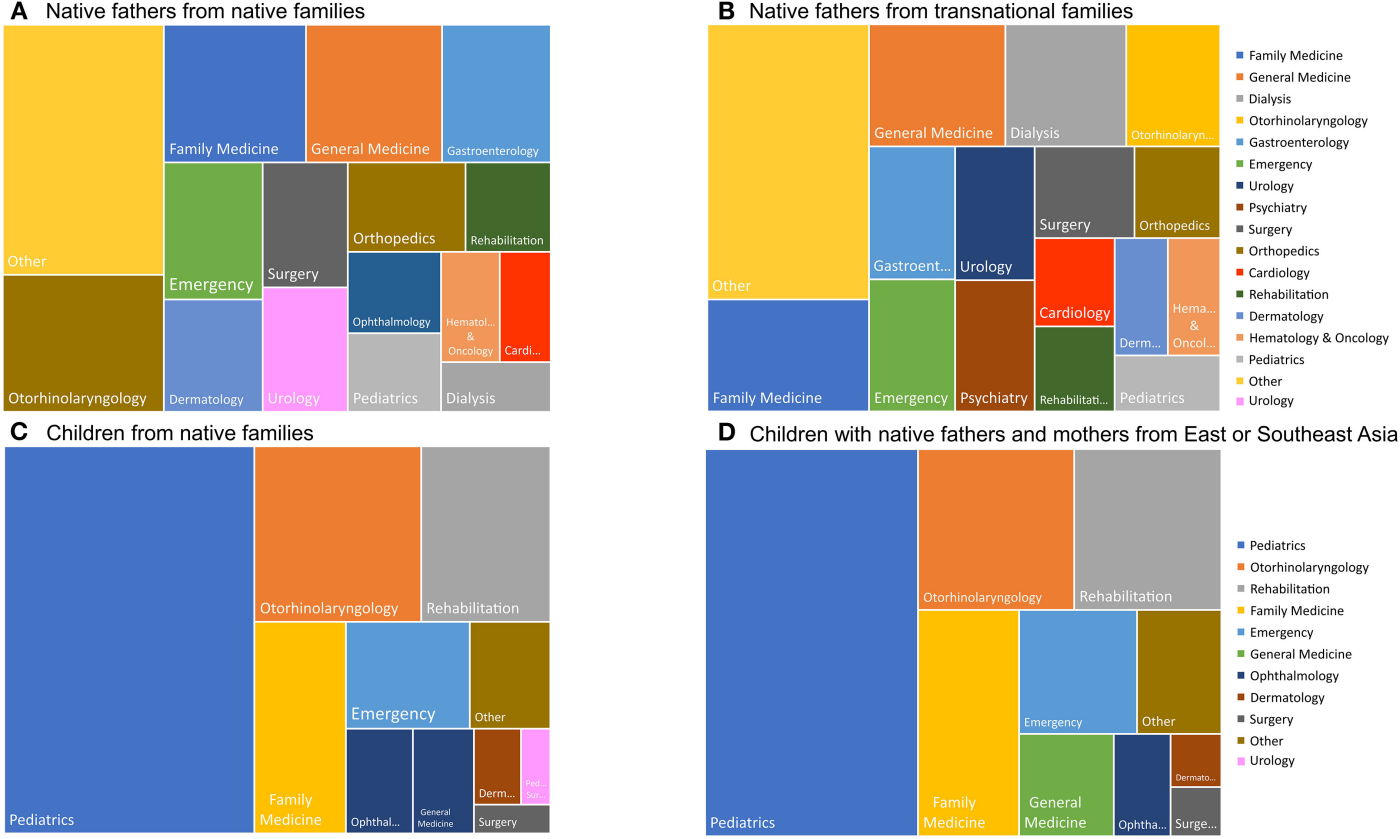
The proportion of total inflation-adjusted medical cost in 2004 to 2017 for top leading medical specialties for fathers from native families **(A)**, native fathers from transnational families **(B)**, children from native families **(C)**, and children from transnational families **(D)**. Medical specialties were included if their explained proportions of the total cost reached at least 2.5%.

**Figure 2:**
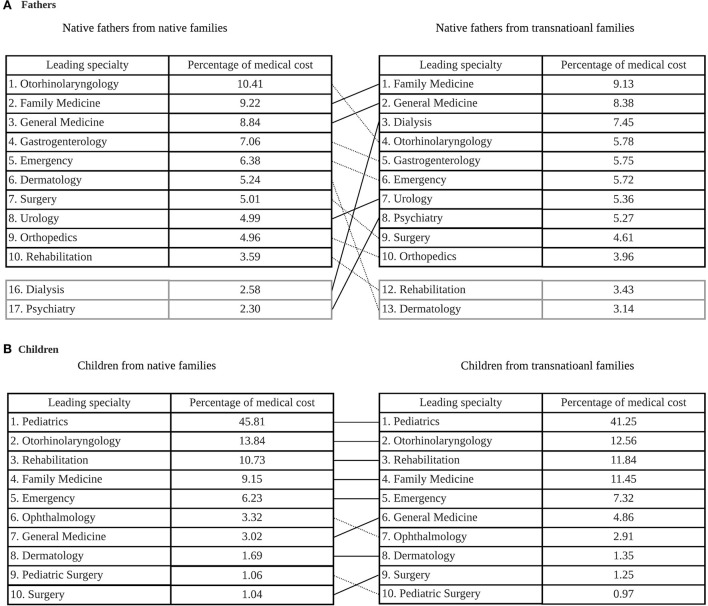
Ten leading medical specialties by total inflation-adjusted medical cost from 2004 to 2017 and percentage difference for fathers from native families and native fathers from transnational families **(A)** and children from native families and children from transnational families **(B)**.

The proportion of the average annual inflation-adjusted medical cost in different medical specialties with regard to the total medical cost between groups is summarized in Figure 1. Generally, the top leading medical cost of specialties was for general medicine, gastroenterology, emergency care, otorhinolaryngology, dermatology, surgery and orthopedics for these fathers. However, specifically compared to fathers from native families, native fathers from transnational families reported higher proportions of medical use in dialysis (7.45 vs. 2.58%) and psychiatry (5.27% vs. 2.30%) but lower proportions in otorhinolaryngology (5.78 vs. 10.41%) and dermatology (3.14 vs. 5.24%). In contrast, unlike their fathers, the proportion and ranking of medical cost in different medical specialties was similar between children from transnational families and children from native families. The leading medical specialties were pediatrics, otorhinolaryngology, rehabilitation, family medicine, emergency care, ophthalmology, and general medicine. These patterns were also reflected in the ranking of medical cost in different top leading medical specialties in Figure 2.

## Discussion

This study demonstrated clear different patterns of health care utilization and different health conditions between native fathers and children from transnational families and fathers and children from native families with full-population data from Taiwan. Different patterns of medical use and costs were observed between fathers and children from transnational families. Specifically, native fathers from transnational families were associated with overall lower medical use but higher medical costs, especially for medical specialties of dialysis and psychiatry, while their children were associated with overall lower medical use and costs. To the best of my knowledge, this is the first study to examine medical use and costs in fathers and children from transnational families. These results provided essential information regarding the medical use and costs in this special subpopulation and public policy implications.

Compared with fathers from native families, lower medical use but higher medical costs and different relative rankings of medical specialties in native fathers from transnational families suggested different patterns of health care utilization and different health conditions between them. Two possible explanations for the inconsistent health utilization (i.e., lower medical use but high medical costs) of native fathers from transnational families. First, it may be the combination of more difficulties in accessing healthcare in native fathers from transnational families and the payment system in the Taiwan National Health Insurance. Although there is high accessibility of healthcare in Taiwan with short waiting times for medical care; individuals can normally reach for consultations on the day that they make an appointment (22), native fathers from transnational families might still have relatively high difficulties in accessing healthcare because they have been reported being more likely to live in rural areas (23) with less medical resources, resulting in lower medical use. This has been supported by a study that the probability of health care utilization were 0.48 to 0.78 times for Taiwanese who resided in rural areas than for those living in areas with the highest urbanization level (24). Furthermore, the Diagnosis-Related Group payments system in Taiwan under the Taiwan National Health Insurance has given additional bonus payments to healthcare providers in rural areas in order to eliminate the rural-urban gap in healthcare infrastructure. Under these circumstances, individuals with similar or lower health care utilization may had higher medical cost.

Second, the other explanation for the inconsistent health utilization is that native fathers from transnational families had more severe disease conditions that required more medical resources in their physician office visits. In addition, higher medical costs for native fathers from transnational families indicated overall poorer health, which is similar to that observed in low socioeconomic status (25) and ethnic minority (26). I observed that different medical uses and costs was related to specific medical specialties, especially chronic health conditions (i.e., dialysis) and mental health, which are consistent with the limited current literature (10–12). Based on female marriage immigrants' reports on their Taiwanese male spouses, the prevalence of disability of their Taiwanese male spouses was 5.6% (11) to 7.7% (12, 14), all of which was higher than the national statistics of 4.5% in Taiwan (27). Because of disability and care needs among native fathers from transnational families, it has been reported that the transnational marriage with female marriage immigrants is a health care workforce strategy of the male spouse's family members, including male spouses and their parent (28); these female marriage immigrants have to take care of the family members' needs, which has been observed in South Korea (29).

Psychiatry is another medical specialty with a relatively higher ranking in native fathers from transnational families than in those from native families, indicating a higher prevalence of mental disorders and more needs for health care in native fathers from transnational families.

Interestingly, I observed that three medical specialties, otorhinolaryngology, dermatology and gastroenterology, were associated with lower medical use and costs in native fathers from transnational families than fathers from native families. There were two possible explanations. First, it may be a reflection of their attitudes toward the medical illness of otorhinolaryngology, dermatology and gastrointestinal problems as minor ailments. They may not be concerned about these minor ailments, resulting in fewer medical help-seeking behaviors. In contrast, they may be more concerned with major medical illnesses from other common medical specialties, such as family medicine, general medicine, and surgery, which have a large portion of health care expenditure. This is partially supported by a study reporting that Taiwanese husbands of female marriage immigrants had poorer health attitudes and health-promoting lifestyles and behaviors (10). Second, the medical costs might have switched from otorhinolaryngology, dermatology and gastroenterology to family medicine and general medicine. In Taiwan, there is no gatekeeper system in primary care, and people who want to seek formal medical care can normally see a specialist on the day that they make an appointment (22). People who have sufficient medical knowledge of their medical conditions can efficiently seek a medical specialist to solve their illness without extra referrals. However, it has been reported that Taiwanese husbands with transnational marriages were more likely to be less educated (11, 30, 31) and have inadequate health literacy (10). As a result, when they had medical illnesses of otorhinolaryngology, dermatology and gastrointestinal problems, they may see family physicians or general physicians instead of otorhinolaryngologists, dermatologists, or gastroenterologists.

Unlike fathers from transnational families, children from transnational families were observed to have lower medical use and costs, with a similar pattern of ranking in leading medical specialties compared with children from native families. A possible explanation is that there may be barriers and unmet needs for receiving medical care in children from transnational families. Because children usually have access to health care services accompanied by their primary caregivers rather than by themselves alone, such barriers may come from the difficulties in accessing health care services among children's primary caregivers. Compared to natives, immigrants had more difficulties accessing health care services, such as unfamiliarity, transportation and differences in language and culture (17), resulting in a lower health care utilization rate (32–35). These results are not only compatible with the current literature but also indicate the possible barriers of access to health care services for children from immigrant families. Another possible explanation is that parents from transnational families might have more conservative attitude of health care utilization; they may only take their children for healthcare visits under serious health conditions. This is partially supported by the data of Organization for Economic Cooperation and Development and World Health Organization, that the number of healthcare visits was lower in the East or Southeast Asian countries of transnational families than that in Taiwan (36). Finally, it is a priority to address the different medical use and costs in transnational marriage families, which can be accomplished by exploring possible barriers to access health care utilization and increasing health literacy to reduce these barriers.

## Limitations

There were some limitations in this study. First, there was a diversity of transnational marriages in Taiwan; although a large proportion of transnational marriages were based on marriage brokerage, some of them were love marriages. It has been suggested that marriages based on a strong bond between couples have positive impacts on physical and mental health (37). However, I identified transnational marriage families based on nationality information from a national birth registered dataset, which is not able to distinguish the different bases of transnational marriages. Some important demographic characteristics (such as type of living, education and other social determinants) which may explain or confound the study findings was not included in the Taiwan National Health Insurance Research Database. In addition, some medical costs cannot be captured in the Taiwan National Health Insurance Research Database. For example, transportation costs and medical unnecessary health care intervention (e.g., cosmetic surgery) are not covered by the Taiwan National Health Insurance (38). Finally, the generalizability of these medical uses and costs to age ranges and other countries is limited. In terms of age ranges, because I used the national birth registered datasets, which were established from 2004 to 2016 and followed to 2017, the age of children enrolled in this study was not > 13 years old, medical use and costs may not cover the common diseases in late adolescence. Similarly, for fathers, age ranges at childbirth are usually between 20 and 40. Because this study only has a 13-year follow-up period, the majority of the fathers would not be older than 55 years, and medical use and costs may not cover the common diseases in late adulthood. In addition, because Taiwan implemented a national health insurance system, individuals' medical use may differ from that in countries where private health insurance is prevalent, such as the United States.

## Conclusions

The first national cohort study is conducted to understand medical use and costs for native fathers and children from transnational families in Taiwan. Native fathers from transnational families were associated with overall lower medical use but higher medical costs, especially for medical specialties of dialysis and psychiatry, while their children were associated with overall lower medical use and costs. These results indicate that different medical use and costs in immigrant families and provide insight to address different health care utilization by exploring the barriers and unmet needs relating to health care utilization in children and fathers from transnational families.

## Data availability statement

The datasets presented in this article are not readily available because the data that support the findings of this study are available from Ministry of Health and Welfare, Taiwan, but restrictions apply to the availability of these data, which were used under license for the current study, and so are not publicly available. Data are however available from the authors upon reasonable request and with permission of Ministry of Health and Welfare, Taiwan. Requests to access the datasets should be directed to Ministry of Health and Welfare, Taiwan.

## Ethics statement

The studies involving human participants were reviewed and approved by China Medical University Hospital. Written informed consent from the participants' legal guardian/next of kin was not required to participate in this study in accordance with the National Legislation and the Institutional Requirements.

## Author contributions

The author confirms sole responsibility for the following: study conception and design, data collection, analysis and interpretation of results, and manuscript preparation.

## Funding

This work was supported by grants from Ministry of Science and Technology [MOST 103-2314-B-002-021-MY3], Taiwan.

## Conflict of interest

The author declares that the research was conducted in the absence of any commercial or financial relationships that could be construed as a potential conflict of interest.

## Publisher's note

All claims expressed in this article are solely those of the authors and do not necessarily represent those of their affiliated organizations, or those of the publisher, the editors and the reviewers. Any product that may be evaluated in this article, or claim that may be made by its manufacturer, is not guaranteed or endorsed by the publisher.
